# Proteomic characterization and cytotoxic potential of proteins from *Cuscuta* (*Cuscuta epithymum* (L.) crude herbal product against MCF-7 human breast cancer cell line

**DOI:** 10.1186/s12906-024-04495-1

**Published:** 2024-05-20

**Authors:** Umaima Akhtar, Yamna Khurshid, Bishoy El-Aarag, Basir Syed, Ishtiaq A. Khan, Keykavous Parang, Aftab Ahmed

**Affiliations:** 1 Biomedical and Pharmaceutical Sciences, Chapman University School of Pharmacy, 9401 Jeronimo Road, Irvine, CA 92618 USA; 2 Biochemistry Division, Chemistry Department, Faculty of Science, Menoufia University, Shebin El-Koom, 32512 Egypt; 3 Jamil-ur-Rahman Center for Genome Research, International Center for Chemical and Biological Sciences, University of Karachi, Karachi, 75270 Pakistan

**Keywords:** Breast cancer, *Cuscuta epithymum*, Protein, Mass spectrometry, MCF-7 cell line

## Abstract

**Background:**

The burden of breast cancer, the second leading cause of death worldwide, is increasing at an alarming rate. *Cuscuta*, used in traditional medicine for different ailments, including cancer, is known for containing phytochemicals that exhibit anticancer activity; however, the bioactivities of proteins from this plant remain unexplored. This study aimed to screen the cytotoxic potential of proteins from the crude herbal product of *Cuscuta epithymum(L.)* (CE) harvested from the host plants *Alhagi maurorum* and *Medicago sativa.*

**Methods:**

The proteins from CE were extracted using a salting-out method, followed by fractionation with a gel filtration chromatography column. Gel-free shotgun proteomics was subsequently performed for protein characterization. The viability assay using MTT was applied to deduce the cytotoxic potential of proteins against MCF-7 breast cancer cells, with further exploration of the effect of treatment on the expression of the apoptotic mediator BCL2-associated X protein (BAX) and B-cell lymphoma protein 2 (BCL-2) proteins, using western blotting to strengthen the findings from the in vitro viability assay.

**Results:**

The crude proteins (CP) of CE were separated into four protein peaks (P1, P2, P3, and P4) by gel filtration chromatography. The evaluation of potency showed a dose-dependent decline in the MCF-7 cell line after CP, P1, P2, and P3 treatment with the respective IC_50_ values of 33.8, 43.1, 34.5, and 28.6 µg/ml. The percent viability of the cells decreased significantly upon treatment with 50 µg/ml CP, P1, P2, and P3 (*P* < 0.001). Western-blot analysis revealed upregulation of proapoptotic protein BAX in the cells treated with CP, P3 (*P* < 0.01), and P2 (*P* < 0.05); however, the antiapoptotic protein, BCL-2 was downregulated in the cells treated with CP and P3 (*P* < 0.01), but no significant change was detected in P2 treated cells. The observed cytotoxic effects of proteins in the CP, P1, P2, and P3 from the in vitro viability assay and western blot depicted the bioactivity potential of CE proteins. The database search revealed the identities of functionally important proteins, including nonspecific lipid transfer protein, superoxide dismutase, carboxypeptidase, RNase H domain containing protein, and polyribonucleotide nucleotidyltransferase, which have been previously reported from other plants to exhibit anticancer activity.

**Conclusion:**

This study indicated the cytotoxic activity of *Cuscuta* proteins against breast cancer MCF-7 cells and will be utilized for future investigations on the mechanistic effect of active proteins. The survey of CE proteins provided substantial data to encourage further exploration of biological activities exhibited by proteins in *Cuscuta.*

**Supplementary Information:**

The online version contains supplementary material available at 10.1186/s12906-024-04495-1.

## Background

*Cuscuta* (Dodders) is an obligate holostemparasitic plant used individually or in combination with other plants as a traditional medicine in Eastern and South Asian countries [1]. *Cuscuta* is known to possess hepatoprotective, anti-HIV, anti-inflammatory, antimicrobial, anticonvulsant, antiproliferative, antioxidant, antiulcer, and antidiabetic activities. These bioactivities are mainly attributed to their phytochemical constituents, such as flavonoids, saponins, sterols/triterpenes, and tannins [2]. For example, the hydroalcoholic extract of *Cuscuta chinensis* was found to have a modest antiproliferative effect on the breast (MCF-7,  IC_50_ ~ 400 µg/ml) and prostate cancer (PC3) cells (IC_50_ ~ 200 µg/ml) [3]. Studies conducted to evaluate the cytotoxic potential of *Cuscuta* have reported that *Cuscuta campestris*, *Cuscuta kotschyana Boiss*, *Cuscuta reflexa*, and *Cuscuta palaestina* species of this genus to have potential anticancer activity against breast cancer, invivo Ehrlich ascites carcinoma, hepatocellular carcinoma, and prostate cancer [4–7]. It has been established that *Cuscuta* acquires its chemical constituents from its host, and the exchange of secondary metabolites, ribonucleic acids (RNAs), and proteins takes place between the host and *Cuscuta* [8]. These plants elicit defense mechanisms in their host, which might result in the synthesis of compounds not found or expressed under normal conditions. For instance, *Cuscuta kotchiana* grown on *Alhagi maurorum* was reported to contain pratensein glycoside, a flavonoid derivative from the host, with significant anti-HIV activity [9].

Additionally, a case study reported that a tincture obtained from *Cuscuta campestris* harvested from *Medicago sativa*, one of the main hosts of *Cuscuta*, had beneficial effects on the recovery of patients with bladder cancer. The study also reported the cytotoxic effect of the product on different cell lines and suggested that the use of *Cuscuta* in combination with the host plant might have a better therapeutic effect [10]. Hence, constituents and/or secondary metabolites expressed as part of the host defense system might be the reason for its better cytotoxic potential than that of other plants. It is important to note that the main patents developed to treat diseases using the *Cuscuta* plant exploited it as a polyherbal product [11].

Cysteine proteinase inhibitors from plants are considered promising antimetastatic and anticancer agents with a better safety profile [12]. Hettenhausen et al. reported the increased production and activity of trypsin proteinase inhibitors in response to multiparasitism in *Cuscut*a [8]. *Cuscuta* produces a pre-pro peptide Cuscutain, a cysteine proteinase. Recently, the pro-peptide segment of Cuscutain was shown to have an inhibitory effect against its enzymatic activity [13]. The proteinase inhibitors found in *Cuscuta* have been studied for parasite plant control, but their therapeutic significance has not been explored. Despite reports of bioactive proteins with pharmacological potential from plants, including parasitic plants and the presence of cysteine proteinases in *Cuscuta* species, there is limited data available for proteins from these species, and to the best of our knowledge, only one study has been conducted to evaluate the bioactive potential of proteins from *Cuscuta* in which the cytotoxic and antioxidant properties of proteins from *Cuscuta europaea*, showed noticeable cytotoxic effects on skin (B-16) and murine mammary breast cancer cell lines (C127) [14].

Based on several reported cases, breast cancer is the most common malignancy responsible for fatalities in women. It is one of the leading causes of death globally, according to the World Health Organization (WHO) [15]. It is immunohistochemically classified into estrogen and progesterone receptor-positive (ER/PR+), human epidermal growth factor receptor-2 positive (HER2+), and triple-negative types. ER/PR + has been diagnosed in approximately 80% of the patients. The phenotypic, molecular, and functional heterogeneity of breast cancer has made its diagnosis and therapy difficult [16]. GLOBOCAN predicted 2.4 million new breast cancer cases, according to demographic changes in 2020, which accounted for 1 out of 4 cancer cases in women [17]. Breast cancer is more prevalent in the industrialized world, but a drastic increase in its incidence rate has been witnessed in developing countries in recent years [18]. Although surgery, hormonal therapy, chemotherapy, and radiation are currently used to treat breast cancer, drawbacks, such as adverse effects, cancer recurrence, high cost, and resistance, limit their effectiveness [19]. Therefore, a search for new therapeutic drug candidates with fewer side effects and better efficacy is necessary.

The research on complementary treatment options for breast cancer revealed that fruits such as grapes and pomegranate possess antiproliferative and apoptotic effects [20, 21]. *Cuscuta* belongs to the *Convolvulaceae* family, the members of which, such as *Convolvulus scammonia* and *Ipomoea batatas* have previously been reported to possess anticancer activity [22]. The molecular phylogenetics research revealed that *Cuscuta* is the closest relative to the *Ipomoea batatas* species [23]; the protein extract (Kunitz-type trypsin inhibitor) and its purified protein (patatin) have previously been found to exert antiproliferative activity against different cancer types [24–28]. These reports encouraged us to evaluate the antiproliferative potential of proteins from the crude herbal product of *Cuscuta epithymum* (L.) (CE) grown on host plants *Alhagi maurorum* and *Medicago sativa* against estrogen-sensitive MCF-7 human breast cancer cell line. *Cuscuta epithymum* (L.) was known to invade 13 crops and recognized for its therapeutic properties in ancient times [29]. In this regard, the crude and partially purified proteins from CE were monitored for their cytotoxicity and effect on the expression of apoptotic mediator proteins. Furthermore, an LC-MS/MS based method has been utilized to identify and characterize proteins. This study will provide the basis for future investigation of the therapeutic potential of proteins from *Cuscuta*.

## Methods

### Extraction of proteins

*Cuscuta epithymum* (L.) (CE) (alfalfa dodder) harvested from host *Alhagi maurorum* and *Medicago sativa* was gifted by Mr. Abbas Isfahanizadeh from Isfahani Medicinal Plants, Sirjan, Iran. The plant material was taxonomically identified by Dr. Hadi Salehirad at the School of Pharmacy,  Tehran University of Medical Sciences, Iran, and registered with herbarium code 7242-TEH. Chapman University’s experimental research policy approved the wet lab research.

The extraction was performed with slight modification using a previously reported method [30]. Before the extraction of proteins, *Cuscuta* tendrils were ground into a fine powder and soaked in 100% hexane (1;6 w/v) for 24 h at room temperature. The defatted sample was passed through Whatman filter paper # 2 and then dried in a fume hood to remove residual solvent. The dried defatted material was then resuspended in phosphate buffer saline (PBS) pH ~ 7 for 24 h with continuous stirring at 4 °C. The homogenous mixture was centrifuged at 10,000 rpm for 30 min at 4 °C. Total soluble proteins in the supernatant were precipitated by ammonium sulfate until 80% saturation was achieved. The amount of ammonium sulfate added to achieve the desired concentration was calculated using an online ammonium sulfate calculator from Encor Biotechnology (www.encorbio.com/protocols/AM-SO4.htm). The proteins in the mixture were pellet down by centrifugation at 10,000 rpm for 30 min at 4 °C and resuspended in PBS. Crude protein of CE tendrils (CP) was dialyzed in tubing [molecular weight cut off (MWCO) ~ 3 kDa] against deionized water. The samples were lyophilized and stored at -20 °C until use.

### Fractionation of proteins

The fractionation of crude protein was performed on a pre-equilibrated Superdex 75 (Hiload 16/60) Prep grade (GE Healthcare) GFC column attached to an AKTA fast protein liquid chromatography (FPLC) system [31]. The column was equilibrated with elution buffer PBS, and lyophilized CP was dissolved in the same before loading onto the column. The elution was carried out at the flow rate of 1 ml/min, and the absorbance was measured at 280 nm. The GFC peaks collected were ultrafiltered to remove salt using Amicon ultra-15 centrifugal filter devices (EMD Millipore Corporation) with a MWCO 3 kDa.

### Estimation of protein concentration

The concentration of proteins in CP and GFC peaks was determined by utilizing a commercially available Coomassie Bradford assay kit (Thermo Fisher Scientific, USA) following the manufacturer’s protocol for 96-well plates. The standard used to quantify proteins in a sample was bovine serum albumin (BSA).

### Cells culture

The estrogen-sensitive human adenocarcinoma MCF-7 breast cancer cell line (ATCC HTB-22) was procured from the American Type Culture Collection (Manassas, VA). The Dulbecco’s Modified Eagle Medium (DMEM) (GIBCO) supplemented with 10% fetal bovine serum (FBS) (GIBCO) and 1% v/v penicillin-streptomycin was used for the maintenance of cells. The normal breast MCF10A cells were maintained in a mammary epithelial cell basal medium (MEBM) and supplemented with mammary epithelial cell growth medium (MEGM) growth factors (Lonza, USA). The cells were incubated at 37 °C with 95% humidity and 5% CO_2_.

### MTT cell proliferation assay

The cytotoxic activity of *Cuscuta* proteins was assessed by 3-(4, 5-dimethylthiazol-2-yl)-2, 5-diphenyl tetrazolium bromide (MTT) assay [32], which has a sensitivity limit of 3000–6000 cells per well. The cells were seeded in a 96 well plate at the density of 3 × 10^4^ cells/ml in 200 µl of total volume per well and placed at 37 °C for 24 h in a 5% CO_2_ incubator. The medium was replaced the next day with 200 µl of DMEM medium containing different concentrations of CP and GFC peaks (25, 50, and 100 µg/ml). The untreated cells that received only the medium were considered control. The cells were incubated for 48 h, and the medium was removed before adding dye. MTT was prepared in the medium (0.5 mg/ml), and 200 µl was added to each well. Plates were allowed to incubate for 4 h in 5% CO_2_ at 37 °C. After incubation, the medium was removed, and 100 µl of dimethyl sulfoxide (DMSO) as a solubilizing agent was added to each well, followed by absorbance measurement at 595 nm with SpectraMax M5 UV/VIS plate reader. The formula used for calculating percent inhibition is as follows:

Percent Inhibition = (O.D. of untreated cells - O.D. of treated cells) x 100/O.D. of untreated cells.

The concentration of protein that decreases viability by 50% (IC_50_) was determined by an online tool, “Quest Graph™ IC50 Calculator.” AAT Bioquest, Inc., https://www.aatbio.com/tools/ic50-calculator [33].

### Western blot analysis

The effect of CE crude protein and fractions P1, P2, and P3 on the protein expression of BCL2-associated X protein (BAX) and B-cell lymphoma protein 2 (BCL-2) was monitored by conducting a western blotting assay [34]. In brief, MCF‑7 cells were seeded onto a 6‑well plate at 1 × 106/ well density and treated with 50 µg/ml of CE crude protein and the fractions for 48 h. After the treatment, the treated and non-treated cells were washed twice in phosphate buffer saline (PBS) and collected. Cell lysates were prepared using the radioimmunoprecipitation (RIPA) buffer extraction reagent (Abcam) supplemented with protease & phosphatase inhibitor cocktail (ThermoScientific, USA). The protein concentrations were determined using a Pierce BCA Protein Assay kit (Thermo Scientific). For immunoblotting, total protein (25 µg) was resolved on 4–12% SDS-PAGE (Mini-Protean TGX Stain-free Gels (Bio-Rad), then transferred onto PVDF membrane (Trans-Blot Turbo Transfer Pack, Bio-Rad, Hercules, CA, USA) by using Trans-Blot Turbo Transfer system (Bio-rad). The membrane was washed with Tris-buffered saline with 0.1% Tween® 20 Detergent (TBST) (0.1%, v/v) three times 10 min each with gentle shaking and blocked in 5% bovine serum albumin (BSA) in TBST. Post blocking, the membrane was then probed overnight at 4 °C with Bax Rabbit mAb (1:1000), BCL-2 Rabbit mAb (1:1000), and Glyceraldehyde 3-phosphate dehydrogenase (GAPDH) Mouse mAb (1:1000) which were acquired from cell signaling Technology (Danvers, MA, USA). The membrane was washed with TBST and incubated with a corresponding HRP-linked antibody for 1 h at room temperature. The membrane was washed again with TBST, and blots were developed using the enhanced chemiluminescence (ClarityTM Western ECL substrate, Bio-Rad). Image J software was used to analyze microphotographs of western blot results.

### In-solution digestion

The lyophilized 1 mg CP and GFC peaks were rehydrated with 100 µl of 6 M urea buffer. The samples were reduced with 5 µl of 200 mM dithiothreitol (DTT), followed by incubation at room temperature for 1 h. Next, 20 µl of alkylation agent, iodoacetamide (200 mM), was added, and the samples were kept for 1 h in the dark at room temperature. The gradual vortexing of samples was done at each step. Further, 20 µl DTT was added to quench the reaction, and the concentration of urea was diluted by adding 775 µl of MilliQ water to the samples. The digestion was carried out by adding TPCK-treated trypsin in the ratio of 1:30 following incubation at 37 °C for 16 h. Acetic acid was added to samples to stop the reaction [35].

### LC-MS/MS data acquisition

The tryptic digested samples were loaded onto BioZen 2.6 μm peptide XB-C18 (pore size 100Å, dimension 150 × 2.1 mm) reverse phase chromatography column (Phenomenex) for LC separation and analyzed by Impact II™ UHR-QqTOF (Ultra-High Resolution Qq-Time-Of-Flight) mass spectrometer (Bruker). The separation of peptides at the flow rate of 0.3 ml/min was achieved using a linear gradient of 0–50% B (0.1% v/v formic acid in acetonitrile) in 45 min.

### Database searching

The acquired raw MS/MS data were processed using ProteinScape software (Bruker) and exported to the mzXML file. PEAKS Studio-X software platform was used for peptide mass fingerprint data search with precursor ion tolerance of 15 ppm and fragment mass tolerance of 0.05 Daltons [36]. The data generated for CP and GFC peaks were searched against a database containing all protein entries for *Cuscuta* in UniProtKB. The score threshold (-10lgP) of > 20, the false discovery rate of < 1%, and carbamidomethyl as fixed while oxidation as the variable modification was selected as database searching parameters in PEAKS-X studio. The mass spectrometry proteomics data have been deposited to the ProteomeXchange Consortium via the PRIDE^57^ partner repository with the dataset identifier PXD044708 and 10.6019/PXD044708.

### Bioinformatics analysis for functional annotation and physicochemical characteristics

UniProtKB was used to retrieve FASTA files for the sequence of all proteins for further analysis. GRAVY score was calculated using an online tool (www.gravy-calculator.de/), and the ProtParam tool from the Expasy server was used to compute molecular weight and isoelectric point. The identified proteins using gene ontology terms were annotated to molecular function, biological process, and cellular component with Blast2GO.

### Statistical analysis

The significance of data was assessed by one-way ANOVA and Dunnett’s post-hoc test using SPSS version 21.0. Results with values *P* < 0.05 were considered statistically significant, whereas *P < 0.05, **P < 0.01, and ***P < 0.001. All data are presented as mean ± standard deviation.

### Data availability

The proteomic data are submitted via the ProteomeXchange dataset, which has been made public via the PRIDE database. ProteomeXchange title: Proteomic Characterization and Cytotoxic Potential of Proteins from *Cuscuta Epithymum* Crude Herbal Product Against MCF-7 Human Breast Cancer Cell Line.

- **ProteomeXchange accession**: PXD044708
- **Project Webpage**: http://www.ebi.ac.uk/pride/archive/projects/PXD044708
- **FTP Download**: https://ftp.pride.ebi.ac.uk/pride/data/archive/2023/08/PXD044708/

## Results

### Extraction and fractionation of proteins from *Cuscuta*tendrils

**Fig. 1:**
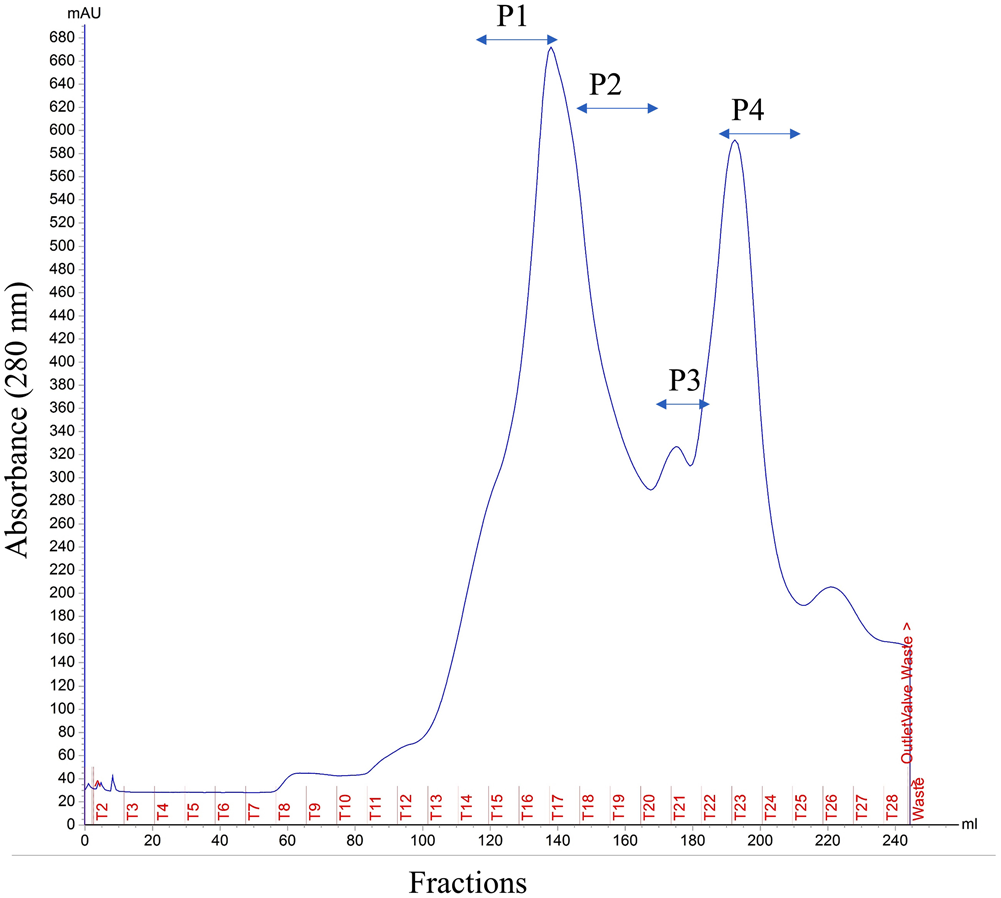
Protein elution profile of *Cuscuta* crude extract on Superdex-75 pg column (GE Healthcare) using PBS as elution buffer. Peaks were labeled with respect to GFC pooled fractions; P1 (T15-16), P2 (T17-18), P3 (T19-21) and P4 (T21-24). The elution was at a 1 ml/min flow rate, and absorbance was measured at 280 nm

Extraction of proteins from the crude herbal product of CE was successfully achieved by applying delipidation, followed by the ammonium sulfate precipitation method. Gel filtration chromatography (GFC) was performed to fractionate crude proteins according to their molecular size distribution. The elution profile from the Superdex-75pg gel filtration chromatography column separated crude proteins into four peaks (Fig. 1). The collected fractions pooled together were named P1 (T15-16), P2 (T17-18), P3 (T19-21) and P4 (T21-24).

### CE proteins exhibit potential cytotoxic activity

**Fig. 2:**
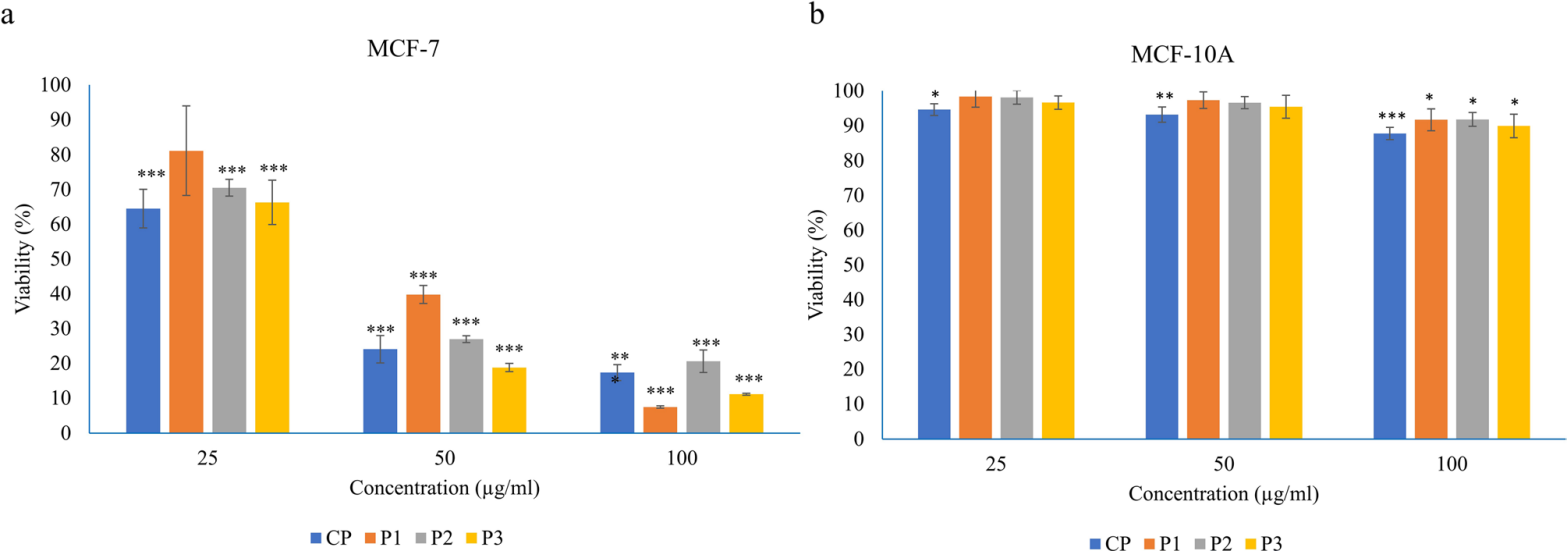
Cytotoxic effect of *Cuscuta* proteins against (**a**) MCF-7 and (**b**) MCF-10 A cell lines. MTT assay assessed proliferation inhibitory activity in dose-response relation at three doses, 25, 50, and 100 µg/ml, after 48 h treatment. Data presented are representative of three independent experiments expressed as percent viability (mean ± SD for triplicate) in CP (crude CE protein), P1, P2, and P3 (GFC peaks) treated cells. The untreated cells were used as a control. The comparison between treated cells and control was evaluated using one-way ANOVA followed by post-hoc Dunnett’s comparison test, whereas ***P < 0.001 **P < 0.01 and *P < 0.05

The crude protein (CP) and GFC peaks were quantified using the Bradford assay. The MTT cell proliferation assay results indicated that proteins in CP and GFC peaks significantly reduced the viability of MCF-7 cells with slight variation. The CP, P1, P2, and P3 were screened further to determine IC_50_ and dose-dependent response at 25, 50, and 100 µg/ml. All peaks inhibited the proliferation of MCF-7 cells in a dose-dependent manner, as shown in Fig. 2a. The percentage of viable cells upon treatment with 50 µg CP, P1, P2, and P3 decreased, and the observed viability became 24.0 ± 6.1, 39.7 ± 8.6, 26.9 ± 3.3 and 18.7 ± 0.97 % (*P* < 0.0001), respectively. The IC_50_ values for CP, P1, P2, and P3 were calculated as 33.8, 43.1, 34.5, and 28.6 µg/ml, respectively (Fig. 2a). Furthermore, CP and GFC peaks showed a nontoxic effect against the MCF10A normal breast cells. Whereas cell viability estimated for the MCF10A at a higher dose (100 µg/ml) was 88.9 ± 1.7 (*P* < 0.001), 91.2 ± 3.1 (*P* < 0.05), 91.3 ± 1.9 (*P* < 0.05), and 89.9 ± 3.4 (*P* < 0.05) % for CP and GFC peaks P1, P2, and P3, respectively (Fig. 2b).

### Effect of *Cuscuta* proteins on BCL2-associated X protein (BAX) and B-cell lymphoma-2 (BCL-2) protein expression

**Fig. 3:**
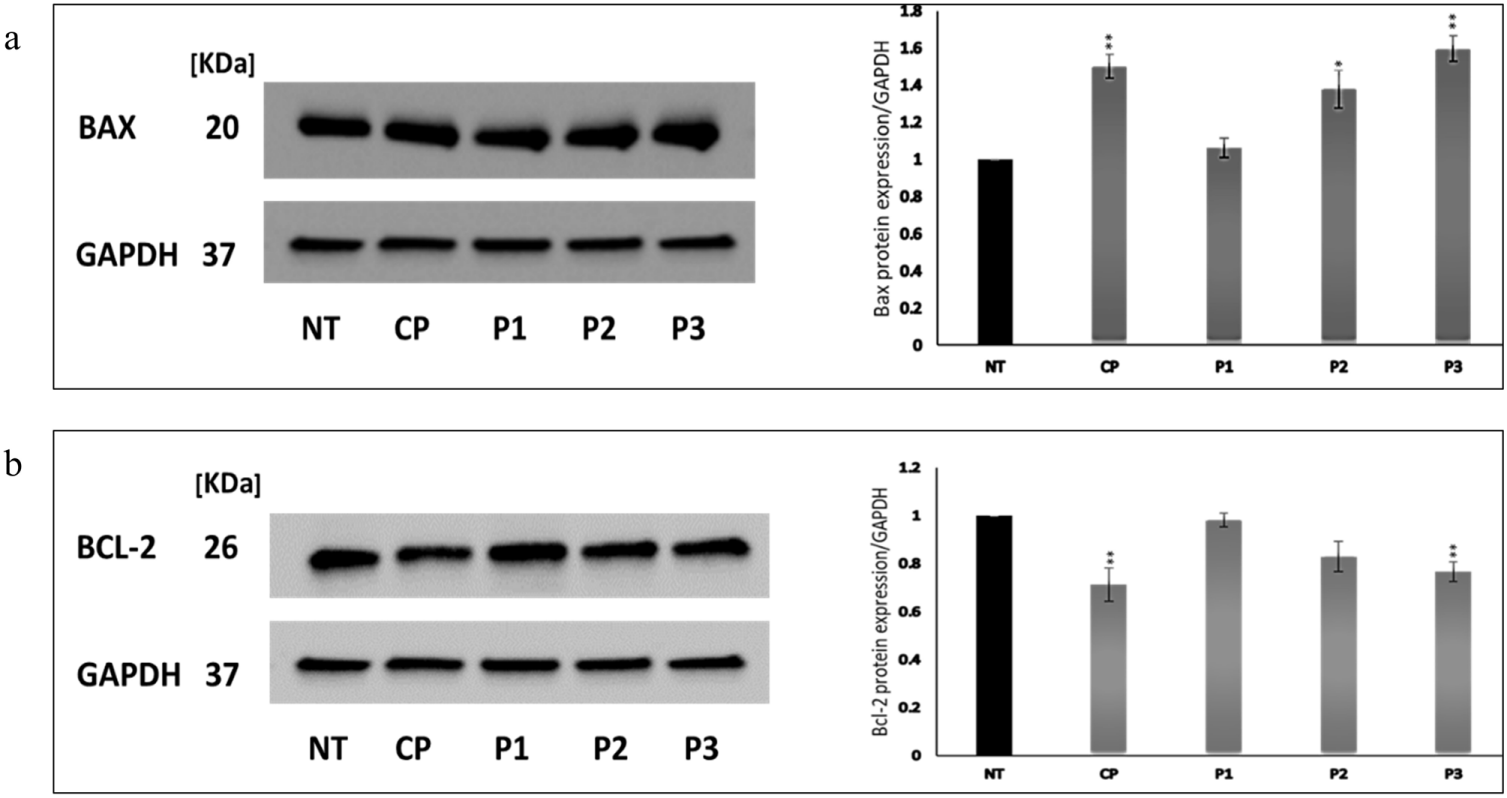
Effect of CE crude protein (CP) and P1, P2, and P3 (GFC peaks) on the BAX and BCL-2 protein expression in MCF-7 cells. Western blot images represent three independent experiments, and statistical analysis is presented for (**a**) BAX and (**b**) BCL-2. Each sample was run in triplicate, and the relative amount of protein was normalized to the GAPDH content in each sample. Data points are represented by the mean ± SD whereas ** *P* < 0.01 and * *P* < 0.05 compared with the untreated control cells (NT). BAX: BCL2 Associated X. GAPDH: Glyceraldehyde 3-phosphate dehydrogenase

The protein expression levels of BAX and BCL-2 in untreated and treated MCF-7 cells with CP and fractions P1, P2, and P3 at 50 µg/ml were examined. Results revealed that CP and fraction P3 significantly decreased the expression of the antiapoptotic protein BCL-2. The treatment with CP and P3 downregulated the protein expression with a fold change of 0.71 and 0.76 (*P* < 0.01), respectively. However, P1 and P2 did not show a significant inhibitory effect on BCL-2 protein expression (Fig. 3a). Conversely, the protein expression of BAX was found to be significantly elevated in MCF-7 cells treated with CP, P3 (*P* < 0.01), and P2 (*P* < 0.05), whereas no significant change appeared in the BAX expression after the treatment with P1. A significant increase of 1.5, 1.4, and 1.6-fold in BAX protein expression was observed after treatment with CP, P2, and P3, respectively, as shown in Fig. 3b. The full-length blots image is shown as supplementary information in Fig. S1-S9.

### Identification of proteins

**Fig. 4:**
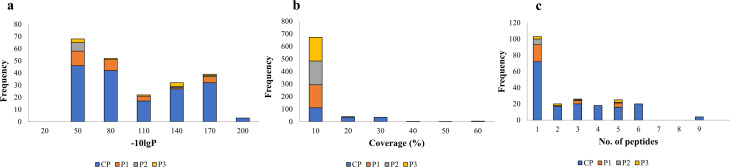
Summary of proteomic data. Acquired LC-MS/MS PMF data was searched against UniProtKB customized database for *Cuscuta* entries using PEAKS Studio-X for CP, P1, P2, and P3, and retrieved results were merged for each sample for graphical representation of the distribution of the frequency of proteins with (**a**) threshold score (-10lgP set to > 20), which is the probability of random peptide matches to the current spectrum, a score of 20 is equivalent to *P* > 0.01; (**b**) percent sequence coverage of proteins by the identified peptides; (**c**) number of identified peptides

The peptide mass fingerprint (PMF) data was generated for CP, and each GFC peak was searched against UniProt-*Cuscuta* entries. The UniProtKB database comprises 262 and 64,709 entries for the *Cuscuta* genus in reviewed (SwissProt) and unreviewed (TrEMBL) entries, respectively. The database search identifies 189 proteins belonging to 66 protein groups. The restricted protein database was utilized only from *Cuscuta* to get a more reliable and convincing list of identified proteins [37]. Most protein groups identified with peptide − 10lgP score of 50 or above (a score of 20 is equal to *P*-value 0.01) (Fig. 4a),10% coverage (Fig. 4b), and at least one unique peptide (Fig. 4c).

The exploration of identified proteins revealed that polyubiquitin and 23 kDa jasmonate-induced protein-like were ubiquitously identified in CP, P1, P2, and P3.

The PMF search for CP retrieved the identity of 57 protein groups, among which heat shock protein (171.31), triosephosphate isomerase (165.62), Ribulose bisphosphate carboxylase large chain (162.14), 23 kDa jasmonate induced like protein (151.89), EF-hand domain-containing protein (150.34) polyubiquitin (149.02) and malate dehydrogenase (147.67.8), were identified with highest scores (Table S1a).

The search results for P1 unveiled the identity of 17 protein groups. Among the proteins in the P1, polyubiquitin and 23 kDa jasmonate-induced like proteins were found to have the highest score of 151.89 and 149.02, respectively, while RNase H domain-containing protein and strictosidine synthase conserved region domain-containing protein identified with a score of ~ 100 followed by carboxypeptidase, which has a score of 74 (Table S1b).

The proteins in P2 were identified as polyubiquitin with a score of 149.02, 23 kDa jasmonate-induced protein-like (130.96) along with the pentacotripeptide repeat region of PRORP domain-containing protein, polyribonucleotide nucleotidyltransferase, and methyltransferase type 11 domain-containing protein with an approximate score of ~ 37.

The retrieved proteins in P3 included polyubiquitin (149.02), histone H2B (132.96), and 23 kDa jasmonate-induced protein-like (130.96). Other proteins identified in P3 were RuBisCO large subunit-binding protein subunit beta chloroplastic, and elongation factor Tu with scores of 74 and 38. (Table S1d).

### Physicochemical properties of identified proteins

**Fig. 5:**
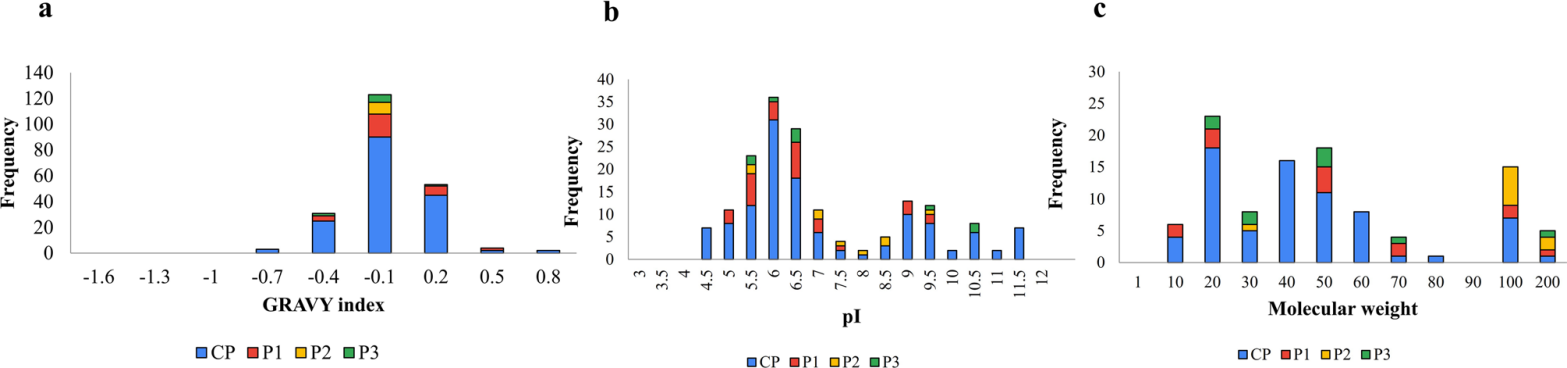
Comparison of physicochemical properties of CE proteins. Full-length protein sequences were used to calculate the physicochemical properties of proteins identified in CP (crude) and GFC peaks. (**a**) Grand average of hydropathicity (GRAVY) scores. Negative values indicate hydrophilic proteins, and positive values indicate hydrophobic proteins, (**b**) theoretical isoelectric point (pI), and (**c**) molecular weight distribution among proteins identified in CP and GFC peaks (P1, P2, P3). Histograms were generated using MS Excel

The grand average of hydropathicity (GRAVY) index estimated for proteins identified in crude protein and GFC peaks represented as histogram depicts proteins in both negative and positive regions in CP, and P1 suggesting the presence of both hydrophilic and hydrophobic proteins, while in P2 and P3, proteins with only negative values were identified (Fig. 5a). The theoretical pI values calculated for proteins identified in CP and GFC peaks showed a bi-modal distribution of *Cuscuta* proteins with respect to pI values in the range of 4–12, as illustrated in Fig. 5b. The rise in frequency of proteins in the acidic pI range of 5-6.5 declines at 7-8.5, and then again, a rise at alkaline pI 9 was observed. Based on molecular weight, *Cuscuta* proteins were observed in the range of 10–200 kDa, with the maximum number of identified proteins with a molecular weight of 20 kDa, as shown in Fig. 5c.

### Functional classification of identified proteins

**Fig. 6:**
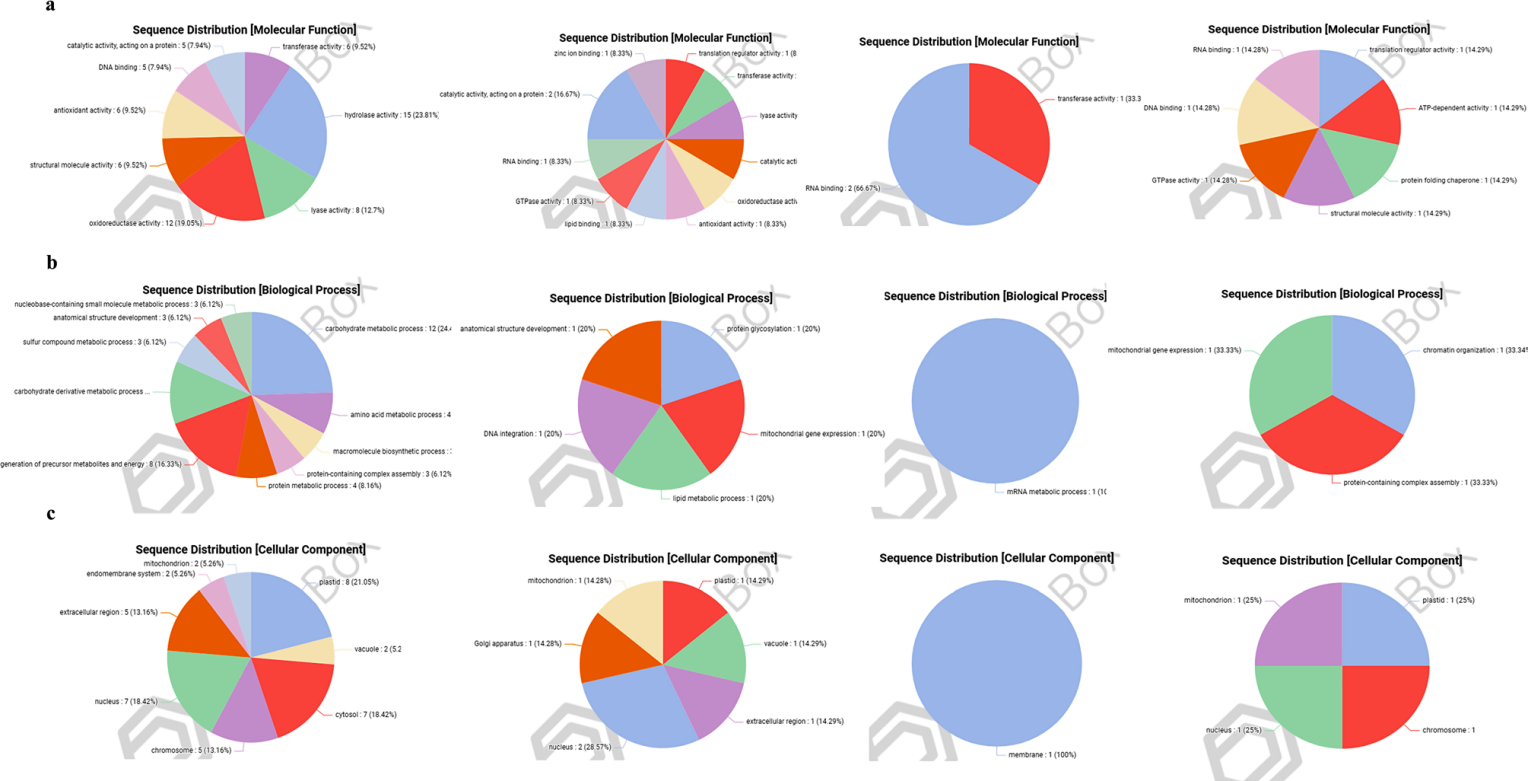
Gene ontology annotation of CE proteins. The sequence of all proteins identified in CP, P1, P2, and P3 was subjected to Blast2GO to classify proteins into GO terms. Pie charts represent the number to a percentage of protein sequences annotated with GO terms derived for categories (**a**) Molecular function, (**b**) Biological process, and (**c**) Cellular component

The proteins in each peak were classified based on their molecular function, involvement in biological processes, and cellular location. CP proteins were identified as those classified with hydrolase activity (23.8%), oxidoreductase (19%), and lyase (12%) activities according to molecular function (Fig. 6a). According to biological process, proteins were found to be involved in carbohydrate metabolic process (24.5%), generation of precursor metabolites and energy (16.3%), and other metabolic processes (Fig. 6b) with respect to biological process, while present mainly in the plastid (21%) chromosome, extracellular region (13%), nucleus and cytosol (18%) concerning cellular localization (Fig. 6c).

The proteins with catalytic activity acting on protein (16%), lyase activity (8%), lipid-binding proteins (8%), along with those associated with antioxidants (8%), and oxidoreductase activities (8%), were present in P1 (Fig. 6a). The proteins identified in P1 were related to protein glycosylation (20%), mitochondrial gene expression (20%), lipid metabolic process (20%), DNA integration, and anatomical structural development (20%) (Fig. 6b). The proteins in P1 were found localized in the nucleus (28.5%), extracellular region (14%), vacuole (14%), plastid (14%) and golgi aaparatus (14%) and other regions (Fig. 6c).

The proteins in P2 were identified with RNA binding and transferase activities (Fig. 6a). They were proposed for taking part in the mRNA metabolic process (Fig. 6b). The only compartment identified to contain P2 protein was the membrane (Fig. 6c).

The proteins associated with protein folding chaperones (14%), ATP dependent (14%), GTPase (14%), and translation regulator activities (14%) were classified in fraction P3. These proteins were involved in mitochondrial gene expression, chromatin organization, and protein containing complex assembly (33%). The portion of proteins in P3 lay in the plastid, nucleus, mitochondrion, and chromosome (25%).

## Discussion

In the current study, to the best of our knowledge, we have reported the biological activity of proteins from the *Cuscuta epithymum* (L.) for the first time. We employed the salting-out method for protein extraction, and then, the separation of proteins was achieved successfully by applying GFC. The crude and GFC peaks were then proceeded for in v*itro* MTT assay to monitor the effect of proteins on the viability of breast cancer cells. The experimental data indicated that proteins in *Cuscuta* crude and GFC peaks suppress the viability of MCF-7 breast cancer cells in a dose-dependent manner, while the viability of normal epithelial breast cancer cells MCF-10 A remains unaffected. The non-cytotoxic effect on estrogen negative MCF-10 A cells suggests that *Cuscuta* proteins produce promising selective cytotoxicity in estrogen receptor positive breast cancer cells [38]. However, the crude and GFC peaks appeared to display little variation in the extent of cytotoxic activity. It might be due to the presence of a single protein, i.e., common to all the fractions that exhibit such a potent activity that even in the presence of other proteins, its effect was inevitable in crude. For instance, the crude and P2 were found to inhibit the viability to a similar extent with IC_50_ values of 33.8 and 34.5 µg/mL. The results demonstrate that the potential cytotoxic activity of proteins in the crude was comparable to proteins in P2 in contrast to GFC peaks P1 and P3. The cytotoxic effect of proteins separated from each other following GFC fractionation was more in P2, and hence, the proteins eluting in P2 were probably responsible for the cytotoxic activity. However, further studies on pure proteins from P2 will be required to draw any conclusion. Nevertheless, the possibility of synergistic action of protein on the viability of MCF-7 cells could not be ruled out, and further investigation is required to identify particular proteins.

The survey of proteins by LC-MS/MS analysis illustrated that proteins identified in CP and P1, namely superoxide dismutase, carboxypeptidase, RNase H domain-containing protein, and nonspecific lipid transfer protein (nsLTP ), were also reported earlier for their anticancer potential from other plants. For instance, the superoxide dismutase identified in P1 and CP was also previously reported in protein fractions from *Corydalis cava*, *Gynura procumbens (Lour.)*, and *Andrographis paniculata* for exhibiting anticancer activity against HeLa, MDA-MB-231 and MCF-7 cancer cell lines, respectively [39–41].

Another protein, carboxypeptidase of bacterial origin, has been found to produce a cytotoxic effect on tumors, which require high folate for growth due to its ability to deplete folate, was also observed in CP and P1. Carboxypeptidases were also identified in Aloe vera, which has been suggested as useful for its chemopreventive potential against various types of cancer [42]. Yates et al. studied that folate supplementation enhanced the growth of the MCF-7 cell line [43]; hence, the folate depleting activity of carboxypeptidase might have a growth inhibitory effect on MCF-7 cells. However, the inhibitor of carboxypeptidase from potatoes was also reported to exert cytotoxic activity by acting as an epidermal growth factor (EGF)/ transforming growth factor alpha (EGF/TGF-alpha) antagonist in the MDA-MB-453 breast cancer cell line [44]. In some reports, a reduction in migration and invasion of MCF-7 cells in the carboxypeptidase knock-down experimental group has been reported [45].

nsLTP from *Brassica campestris* and *Peaganum hermala* has already been demonstrated to possess significant cytotoxic activity towards MCF-7 cells, and other cancer cell lines [46, 47] were also found ubiquitously in CP and P1. A recent study from our lab also described the cytotoxic effect of nsLTP from *Foeniculum vulgare* on MCF-7 cells [48]. Also, nsLTP was one of the proteins reported to be dodder originated mobile proteins [49]. Ribonuclease, considered a non-mutagenic drug in cancer therapeutics, was also identified in CP and P1 [50].

An exoribonuclease polyribonucleotide nucleotidyltransferase (PNPT1), also known as polynucleotide phosphorylase (PNPase), has been reported for promoting cisplatin-induced apoptosis in bladder cancer when overexpressed [51] was identified exclusively in P2. It has also been reported in breast cancer that depletion of PNPT1 results in radioresistance [52].

To gain additional insight into the cytotoxic activity of *Cuscuta* crude extract and the protein fractions, the expression of BAX and BCL-2 in treated MCF-7 cells compared to untreated cells was investigated at a proteomic level. The cells treated with CP extract presented the upregulation of BAX, while decreased expression of BCL-2 was observed in the western blot experiment. These observations suggest that the cytotoxic effect might have involved the apoptotic regulator proteins and possibly occurred via mitochondrial outer membrane permeabilization, which is considered a point of no return for cell death.

The data collected for P1 treated cells suggested that the cytotoxic effect did not occur via targeting expression of apoptotic regulator proteins. Still, they indicated the possible involvement of BH-3 only domain-containing proteins and/or extrinsic pathways. The augmented expression of BAX without affecting BCL-2 protein expression was noticed in the cells treated with P2. We can speculate that an increment in the BAX to BCl-2 ratio might be responsible for the cytotoxic effect observed in MCF-7 cells treated with P2.

In cells treated with P3, significant upregulation in the expression of BAX and downregulation of BCL-2 proteins suggest that P3 induced a cytotoxic effect by regulating apoptotic marker proteins.

It is important to note that polyubiquitin was ubiquitously identified in crude and all the GFC peaks. The previous reports of overexpression of deubiquitinating enzymes in breast cancer cell lines and the presence of polyubiquitin in CP and GFC peaks [53, 54] led us to speculate that the possible release of free ubiquitin might be responsible for cytotoxic activity exhibited by CE proteins. Ubiquitin from mushrooms, such as *Calvatia caelata* and *Ramaria botrytis* has been previously reported for cytotoxic effects against MDA-MB-231 [55] and lung carcinoma A549 cell lines, respectively [56]. Rezvani et al. reported the DNase activity of ubiquitin as one of the putative pathways to induce apoptosis [57]. Extracellular ubiquitin has been previously shown to induce apoptosis and suppress the growth of hematopoietic cells by involving the proteasome degradation pathway [58]. Freiburghaus et al. reported bovine ubiquitin for exhibiting antiproliferative activity against colon cancer and neuroblastoma cells [59]. However, in the HeLa cell, overexpressed ubiquitin was found to accumulate p53 but failed to elicit the apoptotic or growth inhibitory effect [60].

The physicochemical properties of proteins are important characteristics utilized in bioinformatics to predict candidates with anticancer potential. Overall, CP proteins were found in the higher frequency with the pI < 7. According to theoretical molecular mass, crude and all GFC peaks were found to contain 20 kDa proteins with maximum frequency. The molecular weight of 20 kDa is comparable to one of the protein drugs, saporin, which is a 30 kDa ribosome-inactivating protein. The molecular weight and isoelectric point of proteins and peptides have been implemented in bioinformatics tools to determine the penetration and interaction of proteins with negatively charged cancer cell membranes. Previous reports of negatively charged molecules on the surface of bacterial and cancer cell membranes suggest hydrophobicity is an important factor for anticancer drug design. In CP and P1, hydrophilic as well as hydrophobic proteins were observed, while the negative values for P2 and P3 were calculated.

In summary, the cytotoxic activity of CE crude protein extract and proteins collected from GFC fractionation assessed by MTT assay showed dose-dependent inhibition potential. It was observed that CP, P2, and P3 exhibited more cytotoxic activity compared to P1. Also, in western blot analysis, BAX was found upregulated with a decline in BCL-2 in CP, P2, and P3 treated cells. The western blot data suggested different pathways might be involved in exerting cytotoxic effect by CP, P3, P2, and P1. The proteins identified in CP and P1, i.e., superoxide dismutase, nsLTP, and RNase H domain-containing protein, as described above, necessitate further purification and cytotoxic activity evaluation. Also, physicochemical properties, pI, molecular weight, and GRAVY index, which are important features utilized in bioinformatics algorithms for anticancer peptides identification, revealed CE as a source of candidate proteins for further evaluation of biological activities.

## Conclusions

This study uses the parasitic plant CE crude herbal product to characterize the cytotoxic potential of its proteins. The proteins were found to inhibit the viability of the MCF-7 human breast cancer cell line in a dose-dependent manner. The results revealed that *Cuscuta* plant contains proteins with potential cytotoxic activity against breast cancer MCF-7 cells and warrants further elaborated research on the purification of pure proteins and elucidation of the mechanism of cytotoxic activity. The study provided a basis for further exploration of the biological activities of proteins from *Cuscuta*.

### Electronic supplementary material

Below is the link to the electronic supplementary material.

Supplementary Material 1

Supplementary Material 2

## Data Availability

The datasets generated or analyzed during the current study are part of the U.A. doctoral dissertation but are available from the corresponding author upon reasonable request.
