# Supplementary material for: Proteomic characterization and cytotoxic potential of proteins from Cuscuta (Cuscuta epithymum (L.) crude herbal product against MCF-7 human breast cancer cell line
Source: BMC Complement Med Ther. 2024 May 20;24:195. doi: 10.1186/s12906-024-04495-1 (PMC11103822; doi:10.1186/s12906-024-04495-1)
Supplement: Supplementary file 2 — Supplementary Material 2 [file 12906_2024_4495_MOESM2_ESM.pdf]

Supplementary Information:

Proteomic Characterization and Cytotoxic Potential of Proteins from *Cuscuta* (*Cuscuta epithymum* (L.) Crude Herbal Product Against MCF-7 Human Breast Cancer Cell Line

Umaima Akhtar[1,3], Yamna Khurshid[1], Bishoy El-Aarag[1,2], Basir Syed[1], Ishtiaq A. Khan[3], Keykavous Parang[1], and Aftab Ahmed[1]*

1. Biomedical and Pharmaceutical Sciences, Chapman University School of Pharmacy, 9401 Jeronimo Road, Irvine, CA 92618, USA.

2. Biochemistry Division, Chemistry Department, Faculty of Science, Menoufia University, Shebin El-Koom 32512, Egypt

3. Jamil-ur-Rahman Center for Genome Research, International Center for Chemical and Biological Sciences, University of Karachi, Karachi-75270, Pakistan.

* Corresponding author

Aftab Ahmed
Chapman University
School of Pharmacy
9401 Jeronimo Road Irvine, CA 92618 USA
Tel: (714) 516-5465
Fax: (714) 516-5481
aahmed@chapman.edu

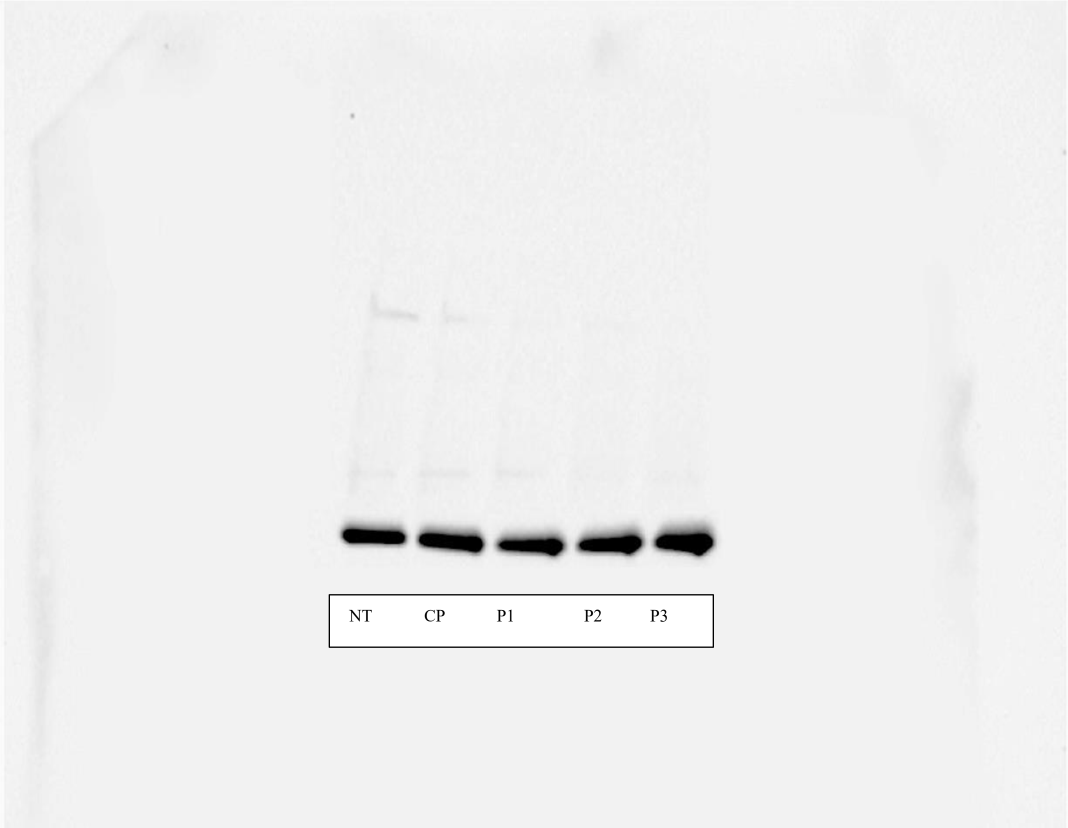

Figure S1: Gel/blots image of Bax protein in non-treated (NT) MCF-7 cells and treated with crude protein (CP) and fractions P1, P2, and P3.

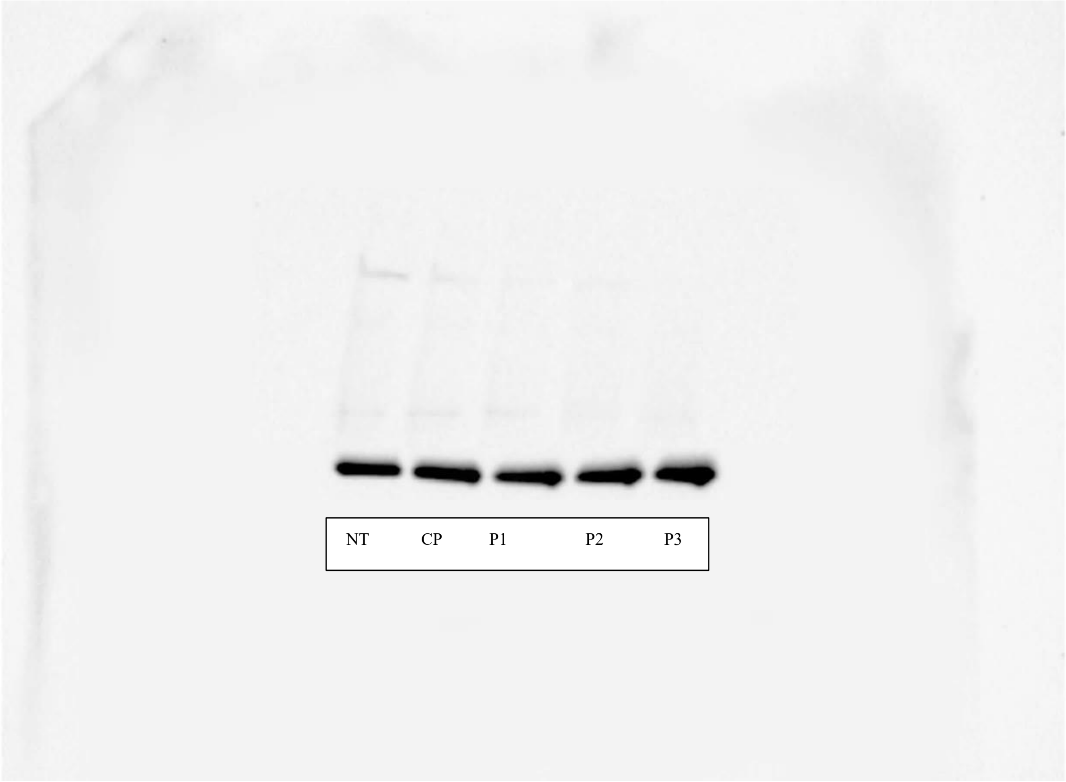

Figure S2: Gel/blots image of Bax protein in non-treated (NT) MCF-7 cells and treated with crude protein (CP) and fractions P1, P2, and P3.

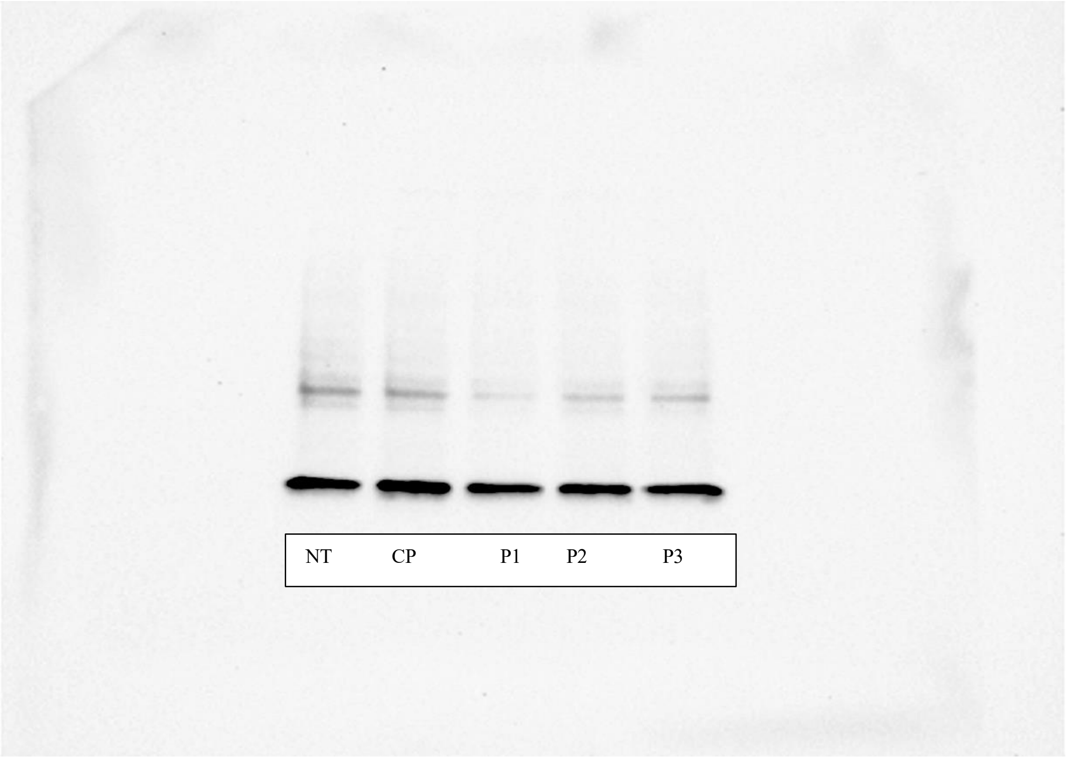

Figure S3: Gel/blots image of Bax protein in non-treated (NT) MCF-7 cells and treated with crude protein (CP) and fractions P1, P2, and P3.

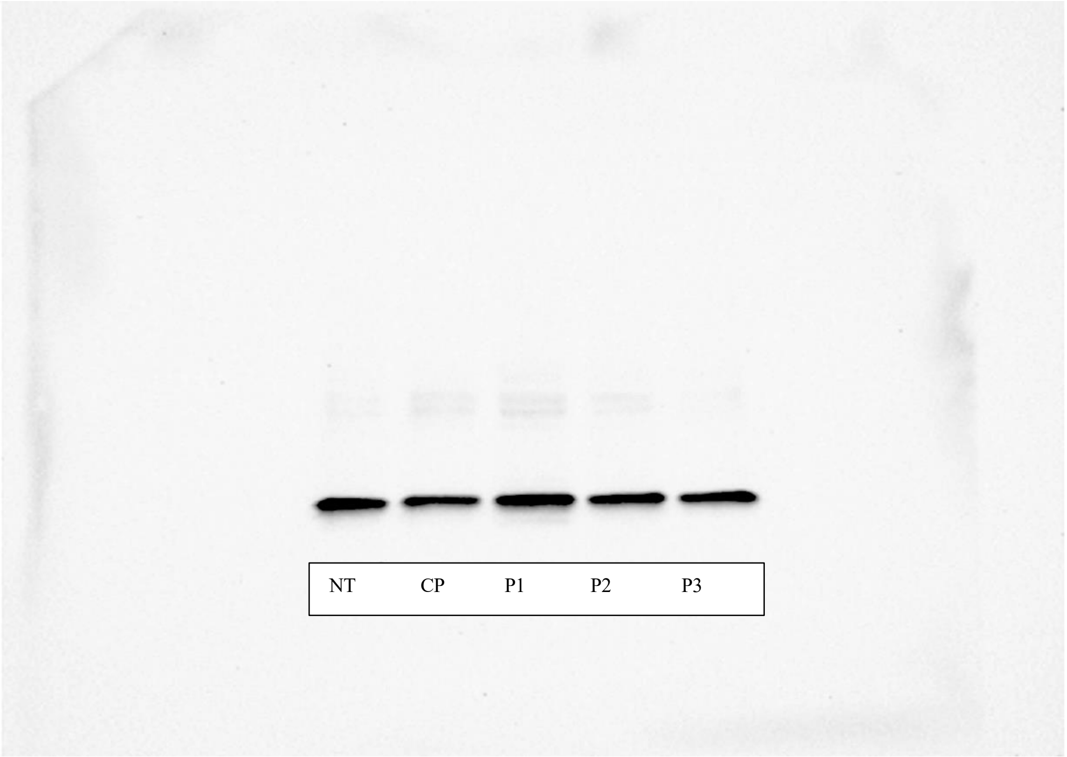

Figure S4: Gel/blots image of Bcl-2 protein in non-treated (NT) MCF-7 cells and treated with crude protein (CP) and fractions P1, P2, and P3.

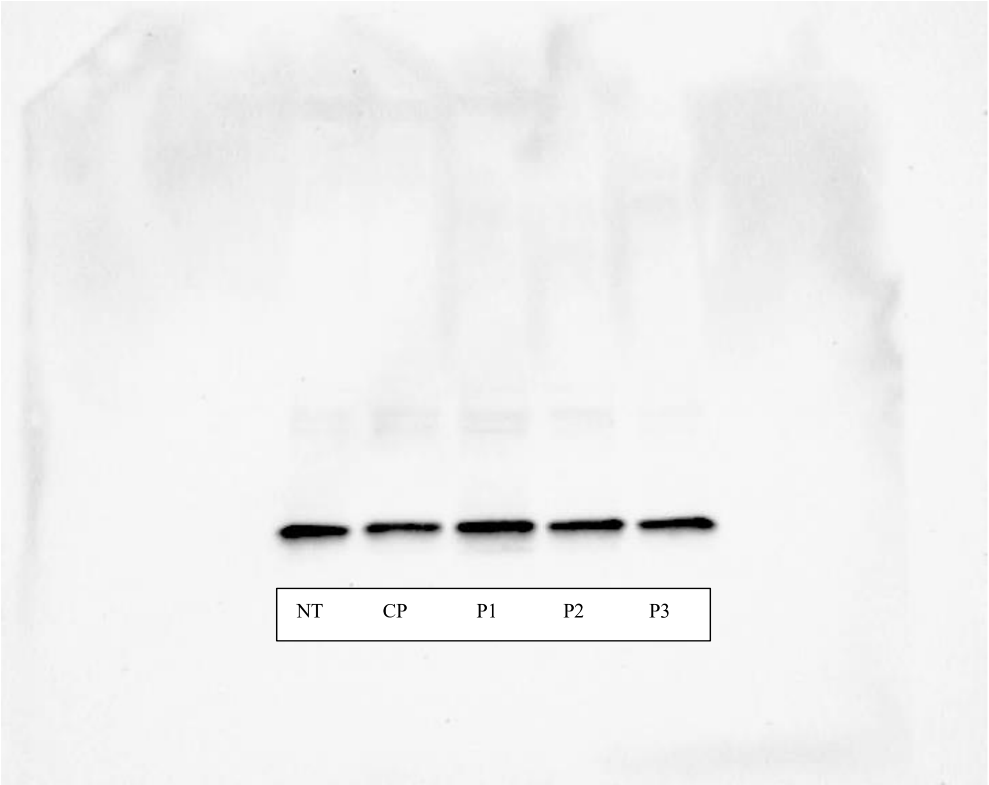

Figure S5: Gel/blots image of Bcl-2 protein in non-treated (NT) MCF-7 cells and treated with crude protein (CP) and fractions P1, P2, and P3.

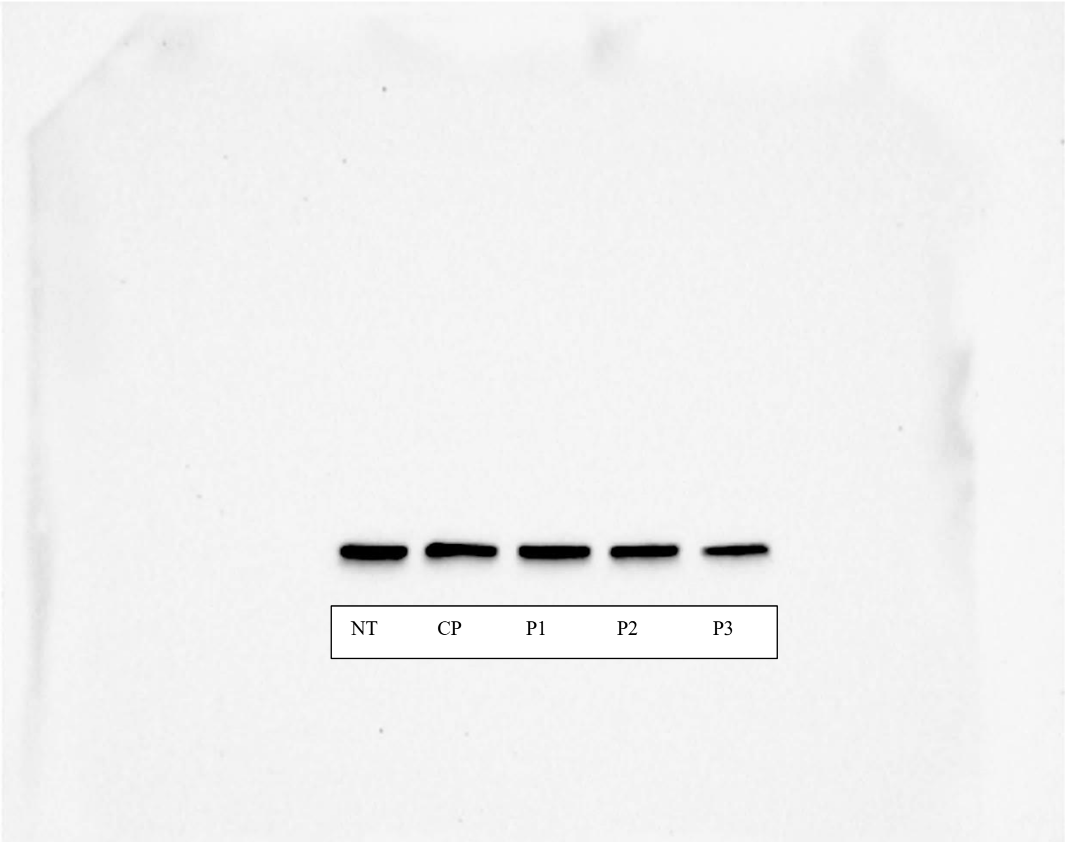

Figure S6: Gel/blots image of Bcl-2 protein in non-treated (NT) MCF-7 cells and treated with crude protein (CP) and fractions P1, P2, and P3.

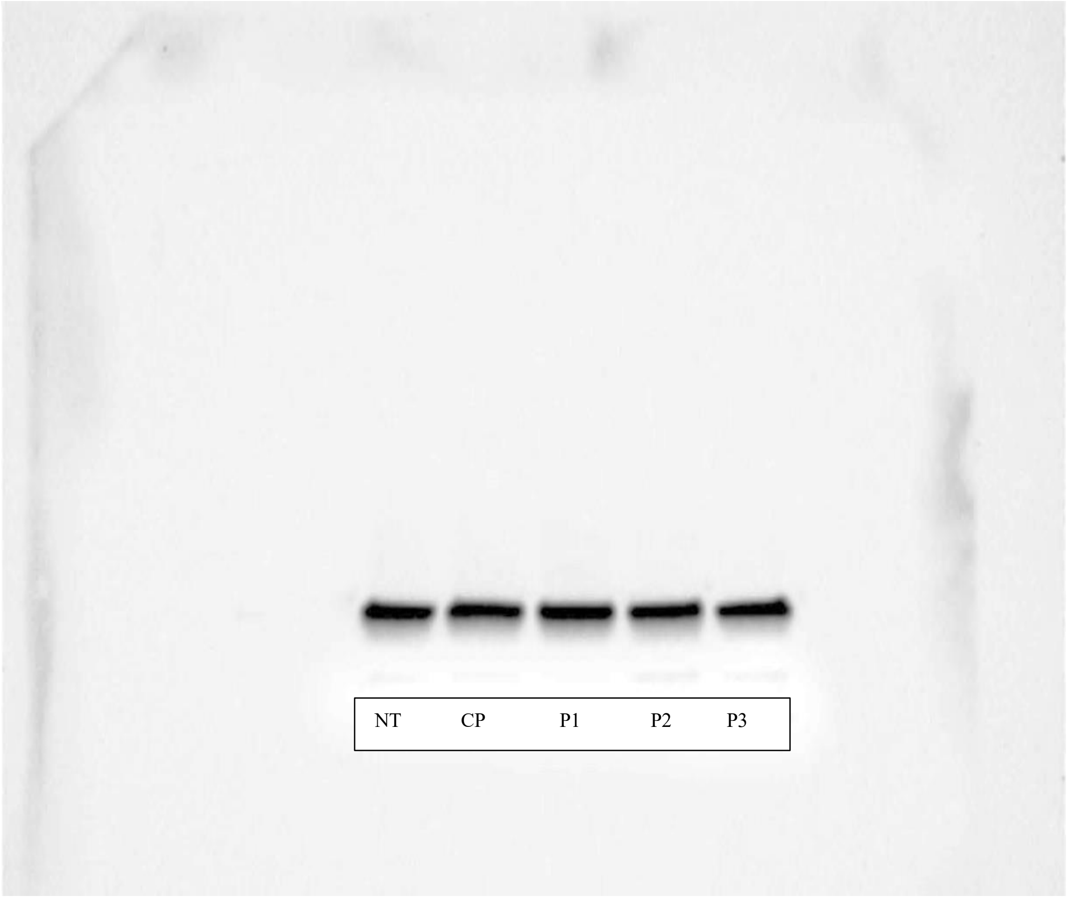

Figure S7: Gel/blots image of GAPDH protein in non-treated (NT) MCF-7 cells and treated with crude protein (CP) and fractions P1, P2, and P3.

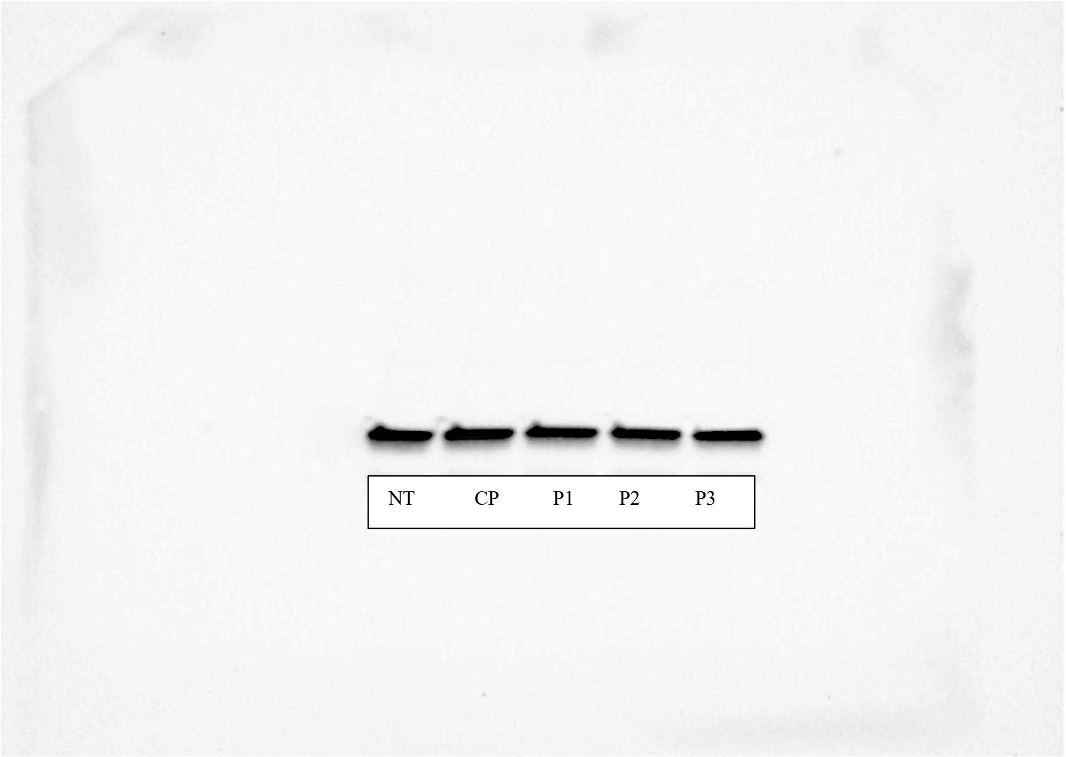

Figure S8: Gel/blots image of GAPDH protein in non-treated (NT) MCF-7 cells and treated with crude protein (CP) and fractions P1, P2, and P3.

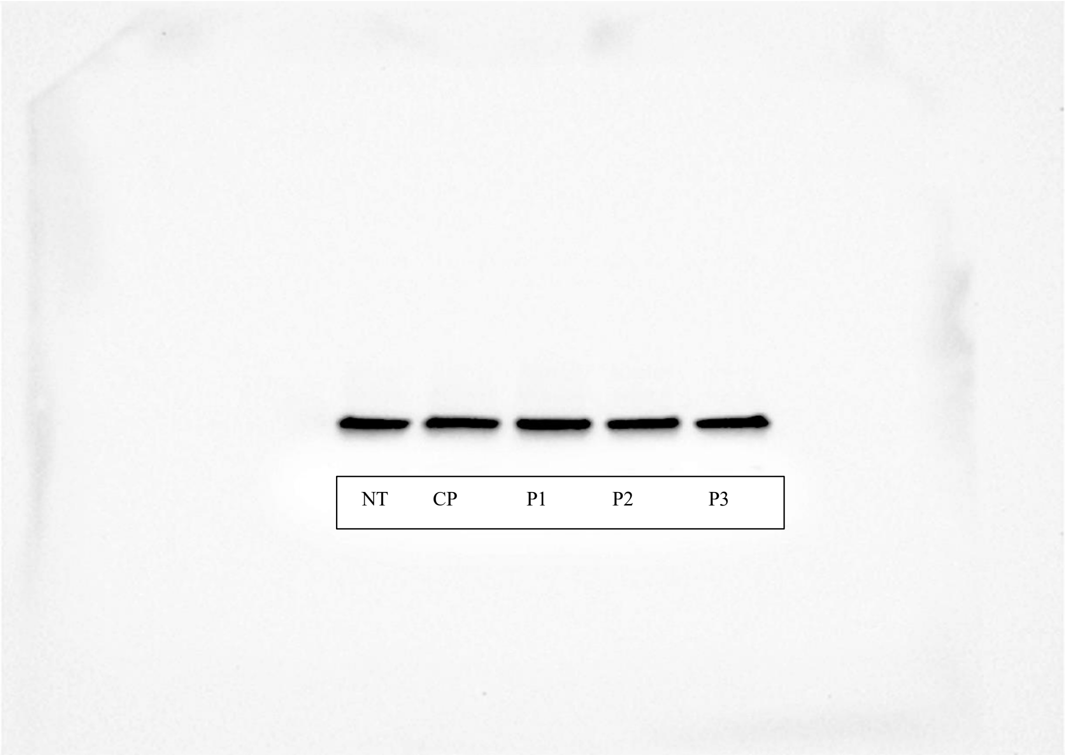

Figure S9: Gel/blots image of GAPDH protein in non-treated (NT) MCF-7 cells and treated with crude protein (CP) and fractions P1, P2, and P3.
